# Genetic diversity in HTLV-1 envelope (gp46), HBZ And LTR region from assymptomatic And TSP/HAM individuals

**DOI:** 10.1186/1742-4690-8-S1-A76

**Published:** 2011-06-06

**Authors:** Aline C A Mota-Miranda, Fernanda Khouri Barreto, Bernardo Galvao-Castro, Luiz C Alcantara

**Affiliations:** 1 Advanced Public Health Laboratory, Gonçalo Moniz Research Center, Oswaldo Cruz Foundation, Salvador, Bahia, Brazil; 2 HTLV Center/ Bahia School of Medicine and Public Health/Bahia Foundation for Science Development, Salvador, Bahia, Brazil; 3 National Cancer Institute, National Institutes of Health, Bethesda, MD, USA

## 

Most HTLV-1 carriers remain infected lifelong without developing any major clinical manifestation. It is known that several antibodies from the sera of HTLV-1-infected individuals can neutralize HTLV-1 transmission. The recently identified HBZ factor acts as a negative regulator of viral transactivation. To better investigate possible differences of gp46, among Health Care (HC) and TSP/HAM individuals, we have performed a point mutation characterization of this component in 146 clones from 10 HTLV-1 infected subjects. The generated sequences were aligned using CLUSTAL X, and edited manually using GENEDOC software to identify possible protein signatures. From the same patient samples, the analyses were performed in the PCR amplified products from HBZ and LTR sequences (n=10) to check the nucleotide sites changes and to perform a phylogenetic analysis, respectively. We had performed the Neighbor-joining and maximum likelihood phylogenetic analysis with PAUP* software. It was possible to identify, at least, 4 exclusive gp46 mutations among HC clones and 6 among TSP/HAM clones. The five common mutations were detected, with statistically significant difference, between HC and TSP/HAM. The overall genetic diversity was of 0.4% and 0.6% for HC and TSP/HAM clones, respectively. The potential protein domain analysis of gp46 showed, mostly, the presence of CK-2 and PKC phosphorylation, N-myristilation and N-glycosilation sites. In the HBZ sequences analysis it was possible to identify 12 mutations, and 11 of them was found in 100% of the generated sequences. The phylogenetic analysis of LTR region demonstrated that all isolates belong to the Transcontinental subgroup of the Cosmopolitan subtype.

